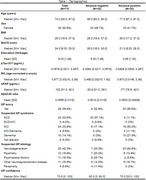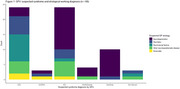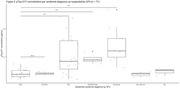# Agreement between Alzheimer's disease blood biomarkers and clinical diagnosis in primary care

**DOI:** 10.1002/alz70856_105489

**Published:** 2026-01-10

**Authors:** Thomas Claessen, Inge M.W. Verberk, David H Wilson, Melchior C. Nierman, Jurgen A. Kooren, Michelle C. Barboure, Wiesje M. van der Flier, Charlotte E. Teunissen, Argonde C. van Harten

**Affiliations:** ^1^ Alzheimer Center Amsterdam, Department of Neurology, Amsterdam UMC, location VUmc, Amsterdam, Netherlands; ^2^ Amsterdam Neuroscience, Neurodegeneration, Amsterdam, Noord‐Holland, Netherlands; ^3^ Neurochemistry Laboratory, Department of Clinical Chemistry, Amsterdam UMC, location VUmc, Amsterdam, Netherlands; ^4^ Quanterix Corp., Billerica, MA, USA; ^5^ Department of Clinical Chemistry, Unilabs / Atalmedial Medical Diagnostic Centers, Amsterdam, Netherlands; ^6^ Amsterdam Neuroscience, Neurodegeneration, Amsterdam, Netherlands

## Abstract

**Background:**

Determining the etiology and severity of cognitive complaints can be difficult in primary care. As a result, diagnosis of Alzheimer's disease (AD) is often inaccurate which may cause diagnostic and therapeutic delay. AD blood biomarkers could support an accurate, timely and non‐invasive AD diagnosis for general practitioners (GPs). We ascertained how many patients likely to be cognitively impaired with evidence of underlying AD were recognized as such by GPs using first data from the ongoing CANTATE‐PC study.

**Method:**

Patients older than 50 years who presented to affiliated Dutch GP practices with cognitive complaints or behavioral changes and gave informed consent were included between December 2023 and December 2024. Demographic and medical data, blood and MoCA scores were collected at home. Participants and informants completed online Amsterdam iADL and ECog‐12 questionnaires and working diagnoses (syndrome and suspected primary etiology) were collected from GPs. Plasma pTau‐217, NfL and GFAP concentrations and the Aβ42/40 ratio were analyzed in one batch using SiMoA (Quanterix, USA). Previously in‐house defined cut‐points were used. We assessed the agreement between GPs’ working diagnosis and positive AD biomarker results in patients who were likely to be cognitively impaired based on a MoCA score <26.

**Result:**

The CANTATE‐PC cohort includes 71 patients so far (median age 74 years, 53.5% female, 54% GP syndrome diagnosis of MCI or dementia (Table 1, Figure 1 and 2)). 42 patients had a MoCA score <26, of which 29 (69%) also had positive AD plasma biomarkers. GPs classified 27/29 patients (93%) as having a syndrome diagnosis of MCI or dementia and 10/29 patients (34%) as having underlying AD.

**Conclusion:**

These are the first results of the ongoing CANTATE‐PC study. Our findings indicate a high agreement between possible cognitive impairment as indicated by a MoCA score <26 and a syndrome diagnosis of MCI or dementia in primary care, but a low agreement between GPs’ etiological diagnosis and abnormal AD blood biomarkers in this group. Our results suggest that AD blood biomarkers may be able to offer additional support to GPs when evaluating the presence of AD.